# Nucleic and Amino Acid Sequences Support Structure-Based Viral Classification

**DOI:** 10.1128/JVI.02275-16

**Published:** 2017-03-29

**Authors:** Robert M. Sinclair, Janne J. Ravantti, Dennis H. Bamford

**Affiliations:** a Mathematical Biology Unit, Okinawa Institute of Science and Technology Graduate University, Onna, Okinawa, Japan; b Department of Biosciences and Institute of Biotechnology, University of Helsinki, Helsinki, Finland; Instituto de Biotecnologia/UNAM

**Keywords:** cotranslational protein folding, more sensitive orphan gene annotation, sequence similarity twilight zone, structure-based viral lineages

## Abstract

Viral capsids ensure viral genome integrity by protecting the enclosed nucleic acids. Interactions between the genome and capsid and between individual capsid proteins (i.e., capsid architecture) are intimate and are expected to be characterized by strong evolutionary conservation. For this reason, a capsid structure-based viral classification has been proposed as a way to bring order to the viral universe. The seeming lack of sufficient sequence similarity to reproduce this classification has made it difficult to reject structural convergence as the basis for the classification. We reinvestigate whether the structure-based classification for viral coat proteins making icosahedral virus capsids is in fact supported by previously undetected sequence similarity. Since codon choices can influence nascent protein folding cotranslationally, we searched for both amino acid and nucleotide sequence similarity. To demonstrate the sensitivity of the approach, we identify a candidate gene for the pandoravirus capsid protein. We show that the structure-based classification is strongly supported by amino acid and also nucleotide sequence similarities, suggesting that the similarities are due to common descent. The correspondence between structure-based and sequence-based analyses of the same proteins shown here allow them to be used in future analyses of the relationship between linear sequence information and macromolecular function, as well as between linear sequence and protein folds.

**IMPORTANCE** Viral capsids protect nucleic acid genomes, which in turn encode capsid proteins. This tight coupling of protein shell and nucleic acids, together with strong functional constraints on capsid protein folding and architecture, leads to the hypothesis that capsid protein-coding nucleotide sequences may retain signatures of ancient viral evolution. We have been able to show that this is indeed the case, using the major capsid proteins of viruses forming icosahedral capsids. Importantly, we detected similarity at the nucleotide level between capsid protein-coding regions from viruses infecting cells belonging to all three domains of life, reproducing a previously established structure-based classification of icosahedral viral capsids.

## INTRODUCTION

Viruses are major players in all known ecosystems (1–7). Viral capsids ensure viral genome integrity by protecting the enclosed nucleic acids (8, 9). Paradoxically, viral genomic diversity is not reflected in virion structural diversity. Viruses typically have only one or two genes encoding capsid proteins due to the size constraints of the genome within the virion. Capsid proteins are used in high copy numbers to form the virion. This has led to the proposal that capsid architectures and capsid protein folds may provide a means of bringing order to the viral universe (10–13). Specifically, it has been suggested that observed similarities between viral coat protein structures may provide a basis for a natural classification of viruses, since the number of types of observed structures is surprisingly low. Each architectural type defines the structural basis of a lineage. Whether this approach can ultimately succeed has been discussed previously at length, but with no clear resolution (14–19), leading us to seek further support from sequence-based analysis and to compare our results with the structural data, although the identification of viral proteins from sequences alone is known to be very challenging due to the overwhelming size and diversity of the virosphere (1, 3, 20, 21). Here, our primary focus will be on the two most established structure-based viral lineages, roughly corresponding to the two supermodules recently identified using gene and genome network analysis (22). One is the PRD1-adenovirus lineage of double-stranded DNA (dsDNA) viruses, whose icosahedral capsids are constructed from major capsid proteins characterized by a vertical double beta-barrel fold (12, 23–27). The other includes capsid proteins from the HK97 lineage (28–30), where the icosahedral dsDNA-containing capsid is constructed from the major coat protein with a long spine helix. We also included myosin, globulin, and sialic acid synthase (NANS) as controls and virus coat proteins from single-stranded RNA (ssRNA) comoviruses that also have the double beta-barrel fold, but in a horizontal orientation in the capsid, unlike PRD1-adenovirus lineage viruses with an upright beta-barrel fold (31).

The fact, that codon choice can and does influence nascent protein folding cotranslationally (32–35) motivated us to search for nucleic acid, as well as amino acid, sequence similarity. We reasoned that strong conservation of protein structure could be reflected in codon choice and therefore may be detectable as coding sequence similarity. Amino acid sequence similarity, even at the level of 88% identity, does not always imply a common fold (36), forcing one to approach the phrase “sequence similarity” from an inclusive point of view, with the expectation that nucleic and amino acid sequence signals complement rather than simply recapitulate each other. The point being made here is that the specific codons possibly influencing protein folding may not necessarily code for the specific amino acids (perhaps as few as 12% [36]) necessary for correct folding. There need not be any overlap, and the sources of detected similarity may be distinct. Therefore, nucleotide and peptide sequence similarity patterns may differ to some extent, as may the equivalent residues in three-dimensional structures, and shedding some light on this is also one of the aims of our study.

The eukaryotic nucleocytoplasmic large DNA viruses (NCLDV) (37) have yielded to amino acid sequence-based phylogenetic analysis (22, 38, 39), but available capsid protein structures suggest that the NCLDV form only one part of a larger grouping, the PRD1-adenovirus lineage (13), which also includes bacterial and archaeal viruses (12, 23–27). In our investigation of nucleotide sequence conservation, we have focused on this relationship.

The capsid proteins we are dealing with are highly divergent, in what is known as the twilight zone of sequence similarity (40, 41). It has been shown that pairwise sequence similarity can provide a more powerful approach than phylogeny-based methods (42) for highly divergent protein sequences. We include three-way sequence similarity for extra sensitivity.

The failure of standard bioinformatics tools, such as BLAST (43), to detect suspected sequence similarities has motivated many teams to shift their focus from sequences to structural information to enhance the evolutionary signals. Unfortunately, such data are still rare and unevenly spread in the case of viruses, particularly so when one considers archaeal viruses (44). Therefore, we have instead asked what we can do to improve the sensitivity of purely sequence-based methods that goes beyond what has been achieved before (41).

## RESULTS

**FIG 1:**
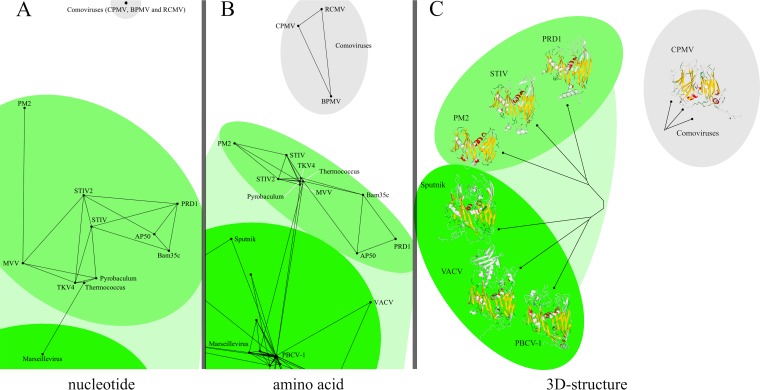
Nucleotide (coding) sequence (A), amino acid sequence (B), and structural similarities (C) detected between PRD1-adenovirus lineage viral coat proteins infecting hosts belonging to all three domains of life. Marseillevirus is a giant virus, belonging to the NCLDV that infects amoebae (39). Bacteriophage PRD1 infects Gram-negative bacteria, while Bam35c and AP50 infect Gram-positive bacteria (12, 26). PM2 is a marine lipid-containing bacteriophage (27). Sulfolobus turreted icosahedral virus 1 (STIV) and Sulfolobus turreted icosahedral virus 2 (STIV-2) infect thermophilic crenarchaeota (25). MVV and TKV4 are euryarchaeal proviruses (24). “Pyrobaculum” stands for a phage major capsid protein identified on an extrachromosomal element of the hyperthermophilic crenarchaeon Pyrobaculum oguniense (45). “Thermococcus” stands for a phage major capsid protein sequence located in the genome of the hyperthermophilic euryarchaeon Thermococcus cleftensis, isolated from a deep-sea hydrothermal sulfide chimney (46). Comoviruses have been included as controls. The black lines indicate detected similarities. All three types of analysis produce the same global connectivity patterns and yet also exhibit differences at the level of specific pairs. These differences highlight the fact that each type of analysis provides an independent window on evolutionary relationships, each emphasizing a different aspect of the same whole. Three-dimensional (3D) structural alignments and trees were produced with the program HSF (31).

**FIG 2:**
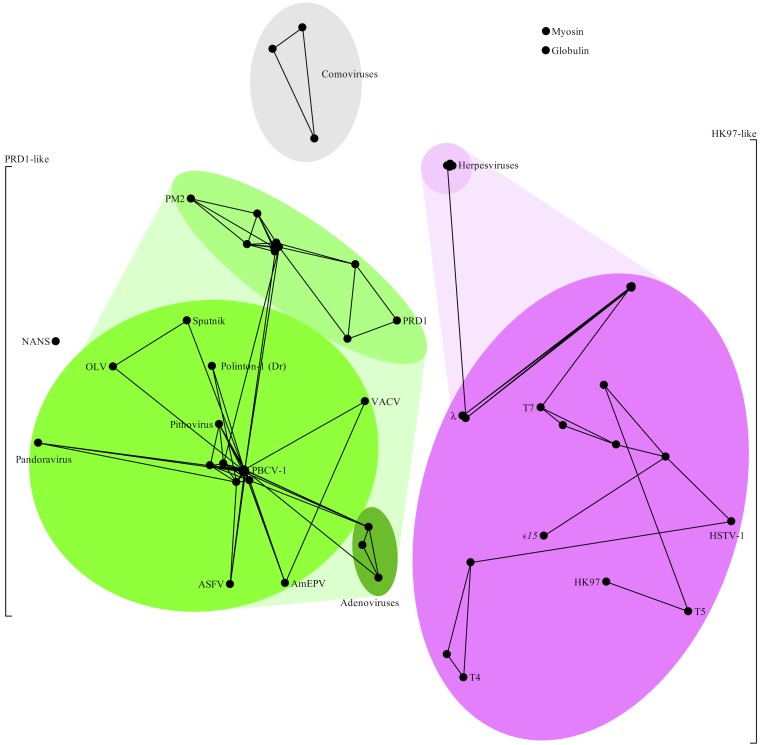
Clustering of virus coat proteins produced by HOSS. Shades of green indicate the PRD1-adenovirus lineage, shades of pink the HK97 lineage, and gray the comoviruses. The two major proposed structure-based viral lineages (PRD1-adenovirus and HK97 lineages) are cleanly separated, providing truly independent, purely sequence-based support for their existence. The comoviruses cluster together but clearly as their own group, with no connections to other lineages, especially to the PRD1-adenovirus lineage. The control protein sequences (myosin, globulin, and NANS) associate neither with each other nor with any of the clusters.

**FIG 3:**
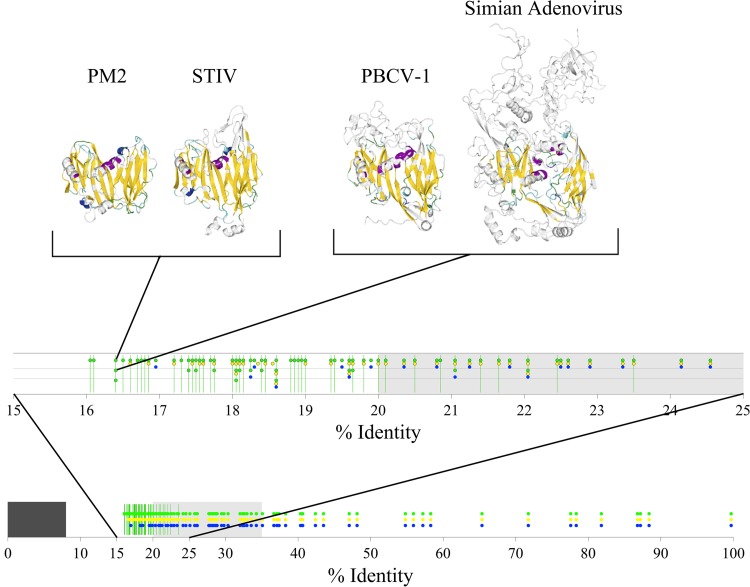
Finding significantly similar sequence pairs from a set of virus coat proteins using HOSS and BLAST. The blue circles indicate the pairs that BLAST search found (E values up to 0.002, as was used to identify the pithovirus sequence [48]). The green circles were obtained by HOSS using both 2-way and 3-way alignments. Percent identity was calculated as the average over alignments using the PAM250 and BLOSUM45 substitution matrices. The yellow circles are raw 2-way alignment results only. The dark rectangle indicates the region from 0% to 8%, where no comparisons apply; the light-gray shading marks the twilight zone (40). The vertical green lines are pairs that HOSS considers significantly similar but BLAST does not. If there are several pairs with the same percent identity, the different pairs are on their own rows. The structure representations on top are aligned, and the secondary-structure elements shown are colored according to the equivalent residues determined by HSF (31).

**FIG 4:**
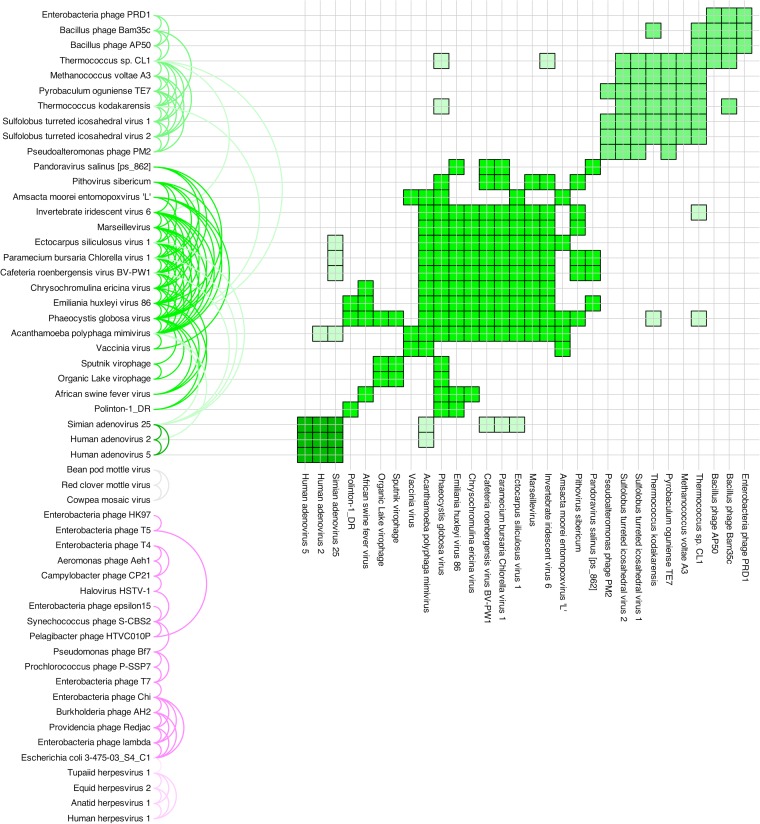
Pairwise significant similarities. Each pair indicated here corresponds to a black line joining two proteins in Fig. 2. The colored semicircular arcs on the left each correspond to one black line in Fig. 2, including all significantly similar sequence pairs. The colors are as in Fig. 2. The matrix on the right shows in clearer fashion exactly which PRD1-adenovirus lineage sequence pairs were found to be significantly similar. The control proteins have been omitted. A similar analysis of the HK97 lineage is presented in Fig. 5.

**FIG 5:**
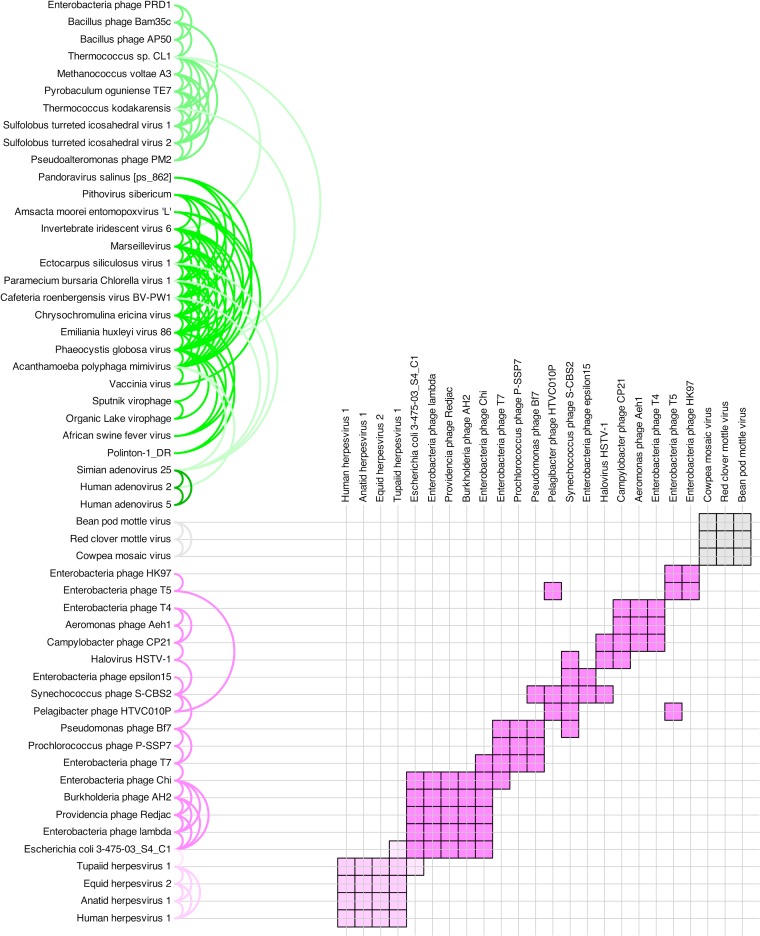
Pairwise significant similarities. Each pair indicated here corresponds to a black line joining two proteins in Fig. 2. The colored semicircular arcs on the left each correspond to one black line in Fig. 2, including all significantly similar sequence pairs. The colors are as in Fig. 2. The matrix on the right shows in clearer fashion exactly which HK97 lineage and comovirus sequence pairs were found to be significantly similar. Note that there are no significantly similar sequence pairs between the HK97 lineage (or the PRD1-adenovirus lineage) and the comoviruses. The control proteins have been omitted.

Before describing our results in detail, it is appropriate to make some general comments, which are intended to allow even a casual reader to understand how Fig. 1 to 5 (and Movie S1 in the supplemental material) are to be understood. First, the most important information in these figures is the lines connecting selected pairs of sequences. These lines exactly reproduce the outcome of our analysis, and the absence of a line between a pair always indicates that our analysis indicated that no line can be drawn, due to a lack of similarity, and never that no attempt was made to find a link. All potential pairs were treated equally. Second, we have attempted to place the sequences in each figure in positions that reproduce the computed dissimilarities, as if dissimilarities could be treated as distances on paper. Unfortunately, it is mathematically impossible to do this without some distortions, just as it is mathematically impossible to print a map of the surface of the Earth on a single piece of paper without distortions (or tearing). For this reason, the reader will notice some pairs that appear, in a figure, to be closer than others and yet do not have a line drawn between them. We ask the reader to focus on the lines as drawn and to excuse these occasional, unavoidable distortions.

### Reproduction of the PRD1-adenovirus structure-based viral lineage from nucleotide sequences alone.

In Fig. 1A, we show that we can detect similarity between the coding sequences for capsid proteins of PRD1-adenovirus lineage viruses infecting all three domains of life, taken from viral (12, 25–27, 39) or host (24, 45, 46) genomes. The extra length of coding sequences with respect to protein sequences (a factor of 3) made it prohibitively expensive to extend the analysis to the full set of viral capsids used, but we believe that the fact that we could detect similarity at all is a considerable achievement and opens the door to future nucleotide sequence similarity analyses. Our achievement is significant because we have detected nucleotide (coding) sequence similarity between proteins whose amino acid sequence similarities have not been detected using standard approaches.

### Reproduction of structure-based viral lineages from protein sequences alone.

We report here (Fig. 1B, 2, 4, and 5; see Movie S1 in the supplemental material), for the first time, the successful detection of signals of protein sequence similarity covering representative viral coat protein sequences from the entire PRD1-adenovirus lineage, including Polinton transposons (47) and the recently resurrected ancient Pithovirus sibericum (48). Furthermore, we are also able to do so for HK97-like viruses, whose lineage is also identified here in accordance with the structure-based hypothesis (10, 11) and in the presence of other viral coat protein sequences (i.e., the comoviruses, belonging to the picornaviruses). Comoviruses are separated from everything else, as are the controls. The same perceived relationships between and within different clusters in Fig. 2 are clearly visible in a three-dimensional animation of the clusters (see Movie S1 in the supplemental material).

We have made a point of being as conservative as possible, to reduce the chance of false positives appearing. The attentive reader will notice that there is only one link visible in Fig. 2 and 5 between herpesviruses and an Escherichia coli phage. It is instructive to examine what this single link represents. Let “E_coli” stand for the “phage major capsid E family protein” annotated in Escherichia coli 3-475-03_S4_C1, “Lambda” for the “capsid component” annotated in Enterobacteria phage lambda, “T_herpes” for the protein T86 annotated in Tupaiid herpesvirus 1, “E_herpes” for the capsid protein annotated in Equid herpesvirus 2, and “A_herpes” for the protein UL19 annotated in Anatid herpesvirus 1 (the details of these four sequences are listed in Table S2 in the supplemental material).

The raw output of our prototype (HOSS, standing for Helsinki-Okinawa sequence similarity) includes three triplets, each of which passes our statistical test for significance (described in Materials and Methods) with respect to both BLOSUM45 and PAM250 substitution matrices. These are <E_herpes/T_herpes/E_coli>, <E_herpes/T_herpes/Lambda>, and <A_herpes/T_herpes/E_coli>. The raw output therefore provides us with potential links between two phage and three herpesvirus sequences. Our conservative analysis requires that a pairing appear twice before a link is considered robust enough to be drawn in Fig. 2 and 5. In this case, only the pair T_herpes/E_coli appears twice. The same approach has been taken to all of our protein sequence data. Every link in Fig. 1B, 2, 4, and 5, as well as Movie S1 in the supplemental material, is backed up by alternatives.

### Sensitivity beyond the twilight zone; identifying a putative pandoravirus capsid protein. (i) Sensitive similarity detection.

In Fig. 3, we show a comparison of our prototype (HOSS) with the commonly used BLAST method. HOSS not only detected plausible similarities with high confidence between protein sequences where BLAST failed, but HOSS was also able to find similarities below 17% protein sequence identity, well beyond what is commonly considered the twilight zone of sequence similarities (40). Since our method does not use any structural information, the pairs of structures illustrated in Fig. 3, which are known to be similar for structural reasons, are an independent confirmation of this level of sensitivity. Without these pairs, we would not have been able to confirm the similarity our method detects between the NCLDV sequences and adenovirus or other PRD1-adenovirus lineage sequences, for example. The BLAST hit with least similarity in Fig. 3, with a pairwise BLAST E value of about 0.002, is between Emiliania huxleyi virus 86 and Invertebrate iridescent virus 6 (see Table S2 in the supplemental material for NCBI accession codes). The associated HOSS score is estimated by HOSS as 0.000000 (see Table S3 in the supplemental material), indicating that HOSS detected very significant sequence similarity (the score is much less than our threshold of 0.0025). Entering either sequence into the NCBI BLAST server with default settings does not retrieve the other. Since it is well known that BLAST hits with E values as large as 0.002 contain a large fraction of false positives, we suggest that this positive control demonstrates the extremely high sensitivity of HOSS.

### (ii) Pandoravirus capsid protein identification.

To test the power and applicability of our new method further, we scanned all annotated open reading frames (ORFs) in the Pandoravirus salinus genome (49), comparing each one to a number of PRD1-adenovirus lineage virus capsid protein sequences. Pandoravirus salinus is a giant virus with a 2.47-Mb genome that encodes 2,556 putative protein-coding sequences (CDSs). About 93% of the CDSs are without recognizable homologs. The few annotated core genes suggest that Pandoravirus salinus is a relative of the NCLDVs, but intriguingly, no capsid protein candidate has been found (22, 49–51). In our scanning, one protein sequence (hypothetical protein ps_862) stood out as having significant pairwise alignments with a number of the capsid proteins tried. We incorporated this pandoravirus protein sequence into our data set and found that it exhibits similarity to NCLDV capsid sequences to an extent that is weaker but comparable (in terms of the number of significant alignments with NCLDV sequences) to the putative capsid protein sequence of NCLDV pithovirus (48) (Fig. 2 and 4; see Table S3 in the supplemental material). We suggest that the hypothetical protein ps_862 of Pandoravirus salinus is a strong major capsid protein candidate and that it would belong to the PRD1 lineage. This suggests, in turn, that our method does have the power to identify orphan genes (21, 52). Experimentally determining the structure of ps_862 is beyond the scope of this work, but we present it because we believe in the scientific value of falsifiable predictions.

## DISCUSSION

Our primary motivation has been to independently support the hypothesis that the structure of viral coat proteins defines a natural classification of viruses (10–13). The hypothesis arises from the observation that virion structure is highly conserved, and it is therefore natural to ask whether this conservation extends to protein or indeed coding sequences. In the absence of both, it would usually be considered to be reasonable to interpret their absence as evidence of structural convergence. Since, however, structural convergence is theoretically achievable with very little amino acid sequence similarity (36), one can imagine structural convergence accompanied by a very low level of sequence similarity, but there is no reason to expect that this would extend to coding sequences to anywhere near the same extent, because the degeneracy of the genetic code typically allows for changes in an encoded amino acid by altering only one or two nucleotides out of three. In such a case, one must expect that a sensitive sequence similarity detection method may find similarity at the protein sequence level but not at coding sequence level. For the same reasons, the detection of extremely weak similarity in both protein and coding sequences by the same method is more likely to be an indication of common descent. The fact that we were able to detect corresponding similarity at both levels has two consequences: it suggests that virion structures are indeed ancient and not a result of convergence, and it shows that protein fold types can be extracted from protein and even nucleotide (coding) sequences. We detected similarity between coding sequences for representative capsid proteins of PRD1-adenovirus lineage viruses infecting all three domains of life. These are expected to have had their most recent common ancestor billions of years ago. We interpret the presence of a detectable nucleotide sequence level similarity signal as a consequence of very strong purifying selection for the PRD1-adenovirus lineage protein fold strongly constraining codon choice, leading to nucleotide sequence conservation.

Our investigations suggest that public databases do not contain intermediate sequences. One can only speculate as to whether this is due to a fragmented occupation of sequence space (53). Such fragmentation could be due to coupling between viruses and their ever-diverging hosts, with last common ancestors between hosts having lived in some cases billions of years ago, or a simple lack of sampling. It is likely that both possible explanations play a role. It is also, for example, well known that several major mass extinctions have occurred in the history of life on Earth, and each one of these would have eliminated both entire classes of hosts and also the associated viruses. Thus, it must be expected that a hypothetical data set of all viral capsid protein sequences that have ever existed may cover sequence space more evenly than the set of all capsid sequences of currently viable viruses. These apparent gaps in sequence coverage, which cannot be bridged using standard methods because they are in the twilight zone, justify the lengths we have gone to in order to recover weak similarities.

Furthermore, our method separates comoviruses clearly from the PRD1-adenovirus lineage, although both viruses possess double beta-barrel folds in their capsid proteins. The difference is in how the capsid proteins are utilized: comoviruses have their capsid proteins formed with horizontally positioned beta-barrels, whereas PRD1-adenovirus lineage capsid proteins are formed with vertically positioned beta-barrels. The fact, that HOSS detected neither protein nor coding sequence similarity between capsid proteins of comoviruses and those belonging to the PRD1-adenovirus lineage demonstrates that it is not confused by this known case of structural convergence (31). This control increases our confidence in the method's ability to separate structural convergence from common descent.

No discussion of specific taxa should distract from the major contribution of our work, which is in showing that these sequences do contain detectable signals and that, in turn, these signals can contribute greatly to discussions concerning possible evolutionary relationships between viruses, as well as the various relationships between structure, amino acid sequence, and coding sequence. Our work is the first to tie structural lineages to sequences so comprehensively.

This is taking us one step further toward bringing order into sequence space and, as more structural data will become available, also to narrowing the gap between linear amino acid and nucleotide sequence information and protein function (folding).

## MATERIALS AND METHODS

We adapted standard (54, 55) computational approaches for the identification of protein sequence similarity, resulting in an approach that is more sensitive than standard bioinformatics tools. We implemented a prototype sequence similarity detection tool (HOSS) for the analysis presented here. In order to achieve the greatest possible sensitivity, we made use of optimal global alignments of two or three sequences at a time, using dynamic programming. The significance of such optimal alignments can be estimated by comparing authentic alignment scores with scores of scrambled sequences of the same compositions and lengths, as suggested before (54, 55), but only now are there enough computing resources for application on such a massive scale. Our computations still required several CPU-years to complete. Using this prototype, we performed statistical analyses of a set of 14 major coat protein and control coding (nucleotide) sequences (see Table S1 in the supplemental material) and 57 major coat protein and control amino acid sequences (see Table S2 in the supplemental material), analyzing 91 plus 3,192 pairs and 364 plus 58,520 triplets over a period of 3 years, using a combination of dedicated desktop computers and high-performance clusters, involving the equivalent of more than 20 3-GHz CPU core years. The prototype method is CPU-intensive due to the large number of sequence combinations and reshufflings, but requirements for other computational resources (memory and disk storage) are negligible.

We wish to emphasize that our primary goal has at all times been to investigate the extent to which structural lineages can be tied to sequences, rather than indulging in bioinformatics or computer science for their own sake. Below, we describe some details of our software implementation, but solely to enable our work to be independently reproduced. We do not wish to propose that we have found the optimal method. What we present below are technical details of the methods that were sufficient to provide us with our results. Readers primarily interested in virology, which is the ultimate focus of our work, may therefore choose to skip this material.

### Implementation details.

Here, we describe our prototype implementation (HOSS) of a classical computational approach (54, 55) in detail, first for pairs of sequences and then for triplets. The description is precise enough for a bioinformatics practitioner to independently reproduce our results. We describe the calculation of dissimilarities, rather than similarities *per se*, because dissimilarity is analogous to distance, and Fig. 1 and 2, as well as Movie S1 in the supplemental material, are most naturally understood in this way. Whether an alignment is considered to be significant is determined by whether the associated dissimilarity is less than or equal to a given dissimilarity threshold. The alignments are used only as a tool to generate dissimilarity values. We do not attempt to perform multiple alignments as an aim in itself.

Let score_2_(*s*_1_,*s*_2_,*M*,*g*) be the optimal alignment score for two sequences, calculated using dynamic programming, for the substitution matrix *M* and gap penalty *g*. For the optimal alignment, each individual column will contain at most one gap (for one sequence) only, and the score for any column with a gap will be −*g*. If the column contains 2 residues (1 for each sequence), then the score for that column is the entry in the matrix *M* corresponding to these 2 residues. The total score for the alignment [this is score_2_(*s*_1_,*s*_2_,*M*,*g*)] is the sum over all the columns of the alignment.

In the first step of our method for pairs, we calculate sc_12_ = score_2_(*s*_1_,*s*_2_,*M*,*g*) and also 10 values of score_2_[scramble(*s*_1_),*s*_2_,*M*,*g*], as well as 10 values of score_2_[*s*_1_,scramble(*s*_2_),*M*,*g*], where “scramble” stands for independent, random reshuffles of the residues of the given sequence, keeping the total length the same. Let *n*_1_ be the number of scores for scrambled versions of sequence 1 that are greater than or equal to sc_12_. Similarly, let *n*_2_ be the number of scores for scrambled versions of sequence 2 that are greater than or equal to sc_12_. If either *n*_1_ or *n*_2_ is greater than 6, then no further computation is performed, and the dissimilarity for the pair of sequences is recorded as follows: diss(*s*_1_,*s*_2_,*M*,*g*) = max(*n*_1_/10,*n*_2_/10).

If computation continues, 100 alignment scores are computed for new scrambled versions of each sequence (200 in total). Let *n*_1_ and *n*_2_ once again stand for the numbers of alignment scores that were greater than or equal to sc_12_. If either *n*_1_ or *n*_2_ is greater than 20, then no further computation is performed, and the dissimilarity for the pair of sequences is recorded as follows: diss(*s*_1_,*s*_2_,*M*,*g*) = max(*n*_1_/100,*n*_2_/100).

The default final step for pairs is to repeat the above for 1,000 new scrambled versions of each sequence. New counts of *n*_1_ and *n*_2_ are made, and the default dissimilarity for the pair of sequences is recorded as follows: diss(*s*_1_,*s*_2_,*M*,*g*) = max(*n*_1_/1,000,*n*_2_/1,000).

For nucleotide sequences, we have used the identity matrix with a gap penalty of 1.

For amino acid sequences, all of the above is repeated for two different substitution matrices (to reduce the probability of false positives, necessitated by the size of the amino acid sequence data set), using the gap penalty of 6 for both, and the default dissimilarity for the pair is recorded as follows: diss(*s*_1_,*s*_2_) = max[diss(*s*_1_,*s*_2_,BLOSUM45,6),diss(*s*_1_,*s*_2_,PAM250,6)].

If this dissimilarity is close to the threshold to be used, new runs with increasingly large numbers (10,000 or 200,000) of rescrambled sequences are performed, and diss(*s*_1_,*s*_2_) is determined on the basis of the longest run. In those rare cases where diss(*s*_1_,*s*_2_) would be zero, we calculate the Z-score using the scores from the longest run executed and estimate diss(*s*_1_,*s*_2_) from the cumulative distribution function for the normal distribution.

For triplets, if a column in the optimal alignment contains no gaps, then the score for that column is the sum of the three matrix entries corresponding to the three ways of pairing the 3 residues in the given column. The scoring of gaps is more involved for triplets than it is for pairs of sequences. If a column contains a single gap, then the score for the column is the matrix entry for the 2 residues minus the gap cost (*g* = 1 or 6). If a column contains two gaps, then the score for the column is minus twice the gap cost (2*g* = 2 or 12).

Just as for sequence pairs, we first compute a small number of alignments with rescrambled sequences, only increasing the number of rescramblings if the results so far indicate a possibly significant result. The number of rescramblings (per sequence, so the total number per 3-way comparison is three times this) begins with 10 and is increased to 100 only if the number of alignment scores greater than or equal to the unscrambled alignment score is less than 7 (for each of the three sequences being compared). Triplets that are then estimated (using Z-scores) to possibly be significant are redone with 1,000, 3,000, 10,000, or 30,000 rescrambles per sequence, with the larger numbers reserved for borderline cases. Several of these cases required on the order of 1 month to complete on a single core.

**FIG 6:**
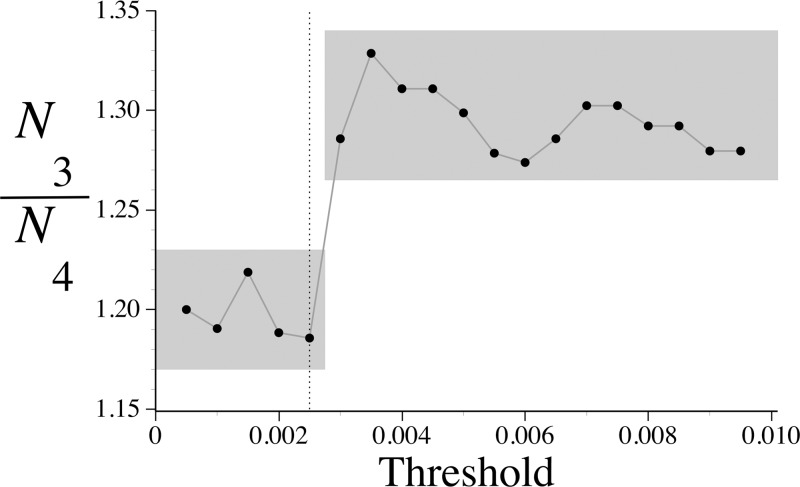
Determination of an appropriate threshold value by looking for a qualitative change in the ratio of numbers of sequence pairs that would inferred to be significant in at least three (*N*_3_) or at least four (*N*_4_) comparisons, allowing the threshold to vary from a starting value close to zero. The vertical dotted line is for the threshold value of 0.0025 that we used.

The threshold value for the large data set of amino acid sequences must be chosen to minimize not only false positives, but also false negatives. Clearly, the number of false negatives will be reduced by simply increasing the choice of threshold value, but there must be a limit. What is to be expected is a qualitative change in the total number of inferred similarities as the threshold is increased beyond a safe level, where the change would be due to an explosion of false positives (40). The threshold value at which this change occurs is the largest value that can be used without fearing a significant number of false positives. Our analysis assigns significant similarity to a pair only if the number of comparisons (pairwise or triplet) in which the given pair appears to be significant is two or more. In order to avoid circular reasoning, we examined instead the ratio of numbers of pairs that are inferred to be significant in at least three (*N*_3_) or at least four (*N*_4_) comparisons. We do indeed see a qualitative change for threshold values greater than 0.0025, as depicted in Fig. 6, where the ratio of *N*_3_ to *N*_4_ is seen to change in a step-like manner. *N*_3_/*N*_4_ jumps upward as the threshold value passes beyond 0.0025, implying a sudden proliferation of pairs supported by only three comparisons. It is for this reason we have chosen 0.0025 as our threshold value.

A list of all significantly similar amino acid sequence pairs with computed dissimilarity values (always second smallest, to reduce the potential impact of false positives) is provided in Table S3 in the supplemental material. The dissimilarities we have computed are not true distances in the geometric sense (they do not satisfy the triangle inequality, meaning they do not add like distances), and this means that any attempt to depict them in a two-dimensional Euclidean space will introduce some distortion. In constructing Fig. 1A and B and 2 and Movie S1 in the supplemental material, we used a monotone increasing function of the raw dissimilarities: *d*(*s*1,*s*2) = 1/{−log[0.990049833749168 diss(*s*1,*s*2)]}, where the constant 0.990049833749168 was chosen to map a raw dissimilarity of 1 (the greatest possible value) to 100 and the logarithm function was chosen to tease apart the very close NCLDV sequences. These sequences often have raw dissimilarity values close to zero. Using a logarithm pulls these apart while still respecting relative dissimilarity (a result of monotonicity). The actual positions of sequences in Fig. 1A and B and 2 were computed by simulated annealing, a standard numerical optimization method involving successive random changes to parameters to find ever better solutions, to find a reasonable approximation or matching between the transformed dissimilarity values and the actual distances on paper between points in Fig. 1A and B and 2. Black lines have been drawn between those pairs of sequences that were judged to be significant on the basis of two or more alignments having raw dissimilarity scores below our threshold value of 0.0025 (see Table S3 in the supplemental material).

## Supplementary Material

Supplemental material
