# Supplementary material for: Nucleic and Amino Acid Sequences Support Structure-Based Viral Classification
Source: J Virol. 2017 Mar 29;91(8):e02275-16. doi: 10.1128/JVI.02275-16 (PMC5375668; doi:10.1128/JVI.02275-16)
Supplement: Supplemental material [file supp_91_8_e02275-16__index.html]

Nucleic and Amino Acid Sequences Support Structure-Based Viral Classification — Supplemental material 

# Nucleic and Amino Acid Sequences Support Structure-Based Viral Classification

## Supplemental material

- Supplemental file 1 -

  Table S1 (Complete nucleotide sequence data set.)

  Table S2 (Complete amino acid sequence data set.)

  Table S3 (Significantly similar amino acid sequence pairs with dissimilarity values below the threshold of 0.0025.)

  Legend to Movie S1

  PDF, 87K
- Supplemental file 2 -

  Movie S1 (Three-dimensional representation of significant similarities and lineages.)

  MOV, 15M
