# Supplementary material for: Nucleic and Amino Acid Sequences Support Structure-Based Viral Classification
Source: J Virol. 2017 Mar 29;91(8):e02275-16. doi: 10.1128/JVI.02275-16 (PMC5375668; doi:10.1128/JVI.02275-16)
Supplement: Supplemental material [file JVI.02275-16_zjv999182474s1.pdf]

TABLE S1: Complete nucleotide sequence data set. The sources of the 14 nucleotide (coding) sequences used in the analysis, downloaded from the NCBI RefSeq or GenBank collections. Unless otherwise indicated, all sequences are complete genomes. For the purposes of nucleotide comparisons, the three comovirus (RNA) sequences serve as controls.

**NCBI Definition**	**Accession**	**Region**
Enterobacteria phage PRD1	NC_001421.2	8595..9782
Bacillus phage Bam35c	NC_005258.1	7699..8769
Bacillus phage AP50	NC_011523.1	7196..8260
Sulfolobus turreted icosahedral virus	NC_005892.1	14942..15979
Sulfolobus turreted icosahedral virus 2	NC_014099.1	14038..15075
Pseudoalteromonas phage PM2	NC_000867.1	5523..6332
Methanococcus voltae A3	NC_014222.1	complement(488365..489390)
Thermococcus kodakarensis KOD1 DNA	NC_006624.1	complement(1188900..1189844)
Pyrobaculum oguniense TE7 extrachromosomal element	NC_016886.1	7060..7986
Thermococcus sp. CL1	NC_018015.1	complement(483758..484702)
Marseillevirus marseillevirus strain T19	NC_013756.1	284716..286149
Cowpea mosaic virus RNA 2	NC_003550.1	1538..2659
Bean pod mottle virus RNA 2	NC_003495.1	1793..2914
Red clover mottle virus RNA 2	NC_003738.1	1509..2636

TABLE S2: Complete amino acid sequence data set. The sources of the 57 amino acid sequences used in the analysis, downloaded from the NCBI RefSeq or GenBank collections. Related protein structures, as PDB entries with used chain ID, with an indication of whether they are from X-ray or Cryo-EM data, are provided where possible. Three non-viral sequences included in the analysis as control protein sequences are marked as controls in the rightmost column.

NCBI Definition	Accession	Region	Length	PDB	Comments	
major capsid protein [Human herpesvirus 1].	NP_044620.1	1055..1374	320 aa			
capsid protein [Equid herpesvirus 2].	NP_042621.1	1049..1381	333 aa			
UL19 [Anatid herpesvirus 1].	YP_003084403.1	1060..1380	321 aa			
T86 [Tupaiid herpesvirus 1].	NP_116435.1	1037..1386	350 aa			
phage major capsid E family protein [Escherichia coli 3-475-03_S4_C1].	KDT88712.1		341 aa			
capsid component [Enterobacteria phage lambda].	NP_040587.1		341 aa			
putative major capsid protein [Enterobacteria phage Chi].	YP_008058174.1		354 aa			
major capsid protein [Burkholderia phage AH2].	YP_006561146.1		342 aa			
capsid protein [Providencia phage Redjac].	YP_006906019.1		357 aa			
major capsid protein [Enterobacteria phage T7].	NP_041998.1		345 aa	3J7V_A	CryoEM	
T7-like capsid protein [Prochlorococcus phage P-SSP7].	YP_214206.1		375 aa	2XD8_A	CryoEM	
major capsid protein [Pseudomonas phage Bf7].	YP_005098192.1		329 aa			
gp23 major head protein [Enterobacteria phage T4].	NP_049787.1	66..521	456 aa			
putative major capsid protein (T4 gp23-like) [Campylobacter phage CP21].	YP_007005149.1		414 aa			
gp24 head vertex protein [Aeromonas phage Aeh1].	NP_944114.1		392 aa			
capsid protein [Halovirus HSTV-1].	YP_008083064.1	107..413	307 aa			
unnamed protein product [Synechococcus phage S-CBS2].	YP_004421492.1		300 aa			
hypothetical protein epsilon15p07 [Enterobacteria phage epsilon15].	NP_848215.1		335 aa	3J40_A	CryoEM	
putative major capsid protein [Pelagibacter phage HTVC010P].	YP_007517707.1		284 aa			
major head protein precursor [Enterobacteria phage T5].	YP_006977.1	160..458	299 aa			
major head subunit precursor [Enterobacteria phage HK97].	NP_037701.1	104..385	282 aa	1OHG_A	X-Ray	
large capsid protein [Red clover mottle virus].	NP_733992.1		376 aa			
large capsid protein [Cowpea mosaic virus].	NP_734001.1		374 aa	1NY7_2	X-Ray	
Large coat protein [Bean pod mottle virus].	NP_734072.1		374 aa	1PGL_2	X-Ray	
PREDICTED: basic 7S globulin [Glycine max].	XP_003521716.1	25..427	403 aa	3AUP_A	X-Ray	Control
myosin [Acetabularia peniculus].	AAB53061.1		933 aa			Control
sialic acid synthase [Homo sapiens].	NP_061819.2		359 aa			Control
major capsid protein [Bacillus phage AP50].	YP_002302529.1		354 aa			
putative protein 18 [Bacillus phage Bam35c].	NP_943764.1		356 aa			
major capsid protein [Enterobacteria phage PRD1].	NP_040692.1		395 aa	1W8X_A	X-Ray	
hypothetical protein CL1_0520 [Thermococcus sp. CL1].	YP_006424522.1		314 aa			
hypothetical protein TK1353 [Thermococcus kodakarensis KOD1].	YP_183766.1		314 aa			
hypothetical protein Mvol_0463 [Methanococcus voltae A3].	YP_003707096.1		341 aa			
coat protein [Sulfolobus turreted icosahedral virus 1].	YP_025022.1		345 aa	3J31_A	CryoEM	
hypothetical protein STIV2_A345 [Sulfolobus turreted icosahedral virus 2].	YP_003591107.1		345 aa			
putative Major Capsid Protein [Pyrobaculum oguniense TE7].	YP_005261453.1		308 aa			

major capsid protein P2 [Pseudoalteromonas phage PM2].	NP_049903.1		269 aa	2VVF_A	X-Ray	
V20 [Sputnik virophage].	YP_002122381.1		595 aa	3J26_A	CryoEM	
major capsid protein [Organic Lake virophage].	ADX05770.1		575 aa			
major capsid protein [Marseillevirus].	YP_003407071.1		477 aa			
major capsid protein [Cafeteria roenbergensis virus BV-PW1].	YP_003969975.1		506 aa			
capsid protein 1 [Acanthamoeba polyphaga mimivirus].	YP_003986929.1		593 aa			
trimeric virion coat protein [Vaccinia virus].	AGJ91296.1		551 aa	2YGB_A	X-Ray	
rifampicin resistance protein [Amsacta moorei entomopoxvirus 'L'].	NP_064904.1		567 aa			
EsV-1-116 [Ectocarpus siliculosus virus 1].	NP_077601.1		476 aa			
putative major capsid protein [Emiliania huxleyi virus 86].	YP_293839.2		496 aa			
274L [Invertebrate iridescent virus 6].	NP_149737.1		467 aa			
major capsid protein MCP [Phaeocystis globosa virus].	YP_008052475.1		519 aa			
Major capsid protein [Paramecium bursaria Chlorella virus 1].	NP_048787.1		437 aa	1M3Y_A	X-Ray	
Major capsid protein [Chrysochromulina ericina virus].	A7U6E7.1		584 aa			
structural protein p72 [African swine fever virus].	NP_042775.1		646 aa			
hexon [Simian adenovirus 25].	AP_000313.1		933 aa	2OBE_A	X-Ray	
hexon [Human adenovirus 2].	CAC67477.1		968 aa	1P2Z_A	X-Ray	
hexon protein [Human adenovirus 5].	AAW65514.1		952 aa	3IYN_A	CryoEM	
divergent major capsid protein [Pithovirus sibericum].	YP_009001361.1		479 aa			
hypothetical protein ps_862 [Pandoravirus salinus].	YP_008437274.1		382 aa			
Polinton-1_DR (Zebrafish DNA sequence from clone DKEY-230I11 in linkage group 23, complete sequence.)	CR759764.7	complement(93467..94774)	1308 bp (436 aa)			Ref. (49)

Sinclair, Ravantti & Bamford -- Nucleic and amino acid sequences support structure-based viral classification

TABLE S3: Significantly similar amino acid sequence pairs with dissimilarity values below the threshold of 0.0025. In order to reduce any effect of false positives, the dissimilarity values provided here are the second-smallest in each case, in the spirit of a trimmed mean.

Sequence 1	Sequence 2	Dissimilarity
Human herpesvirus 1	Equid herpesvirus 2	0
Human herpesvirus 1	Anatid herpesvirus 1	0
Human herpesvirus 1	Tupaiid herpesvirus 1	0
Equid herpesvirus 2	Anatid herpesvirus 1	0
Equid herpesvirus 2	Tupaiid herpesvirus 1	0
Anatid herpesvirus 1	Tupaiid herpesvirus 1	0
Tupaiid herpesvirus 1	Escherichia coli 3-475-03_S4_C1	0.001691
Escherichia coli 3-475-03_S4_C1	Enterobacteria phage lambda	0
Escherichia coli 3-475-03_S4_C1	Enterobacteria phage Chi	0
Escherichia coli 3-475-03_S4_C1	Burkholderia phage AH2	0
Escherichia coli 3-475-03_S4_C1	Providencia phage Redjac	0
Enterobacteria phage lambda	Enterobacteria phage Chi	0
Enterobacteria phage lambda	Burkholderia phage AH2	0
Enterobacteria phage lambda	Providencia phage Redjac	0
Enterobacteria phage Chi	Burkholderia phage AH2	0
Enterobacteria phage Chi	Providencia phage Redjac	0
Enterobacteria phage Chi	Enterobacteria phage T7	0.0019
Burkholderia phage AH2	Providencia phage Redjac	0
Enterobacteria phage T7	Prochlorococcus phage P-SSP7	0
Enterobacteria phage T7	Pseudomonas phage Bf7	0
Prochlorococcus phage P-SSP7	Pseudomonas phage Bf7	0.000012
Pseudomonas phage Bf7	Synechococcus phage S-CBS2	0.00141
Enterobacteria phage T4	Campylobacter phage CP21	0
Enterobacteria phage T4	Aeromonas phage Aeh1	0
Campylobacter phage CP21	Aeromonas phage Aeh1	0.0006
Campylobacter phage CP21	Halovirus HSTV-1	0.0021
Halovirus HSTV-1	Synechococcus phage S-CBS2	0.002
Synechococcus phage S-CBS2	Enterobacteria phage epsilon15	0.002
Synechococcus phage S-CBS2	Pelagibacter phage HTVC010P	0.00118
Pelagibacter phage HTVC010P	Enterobacteria phage T5	0.001333
Enterobacteria phage T5	Enterobacteria phage HK97	0.000667
Red clover mottle virus	Cowpea mosaic virus	0
Red clover mottle virus	Bean pod mottle virus	0
Cowpea mosaic virus	Bean pod mottle virus	0
Bacillus phage AP50	Bacillus phage Bam35c	0.0002
Bacillus phage AP50	Enterobacteria phage PRD1	0.0002
Bacillus phage AP50	Thermococcus sp. CL1	0.0018
Bacillus phage Bam35c	Enterobacteria phage PRD1	0.00058
Bacillus phage Bam35c	Thermococcus sp. CL1	0.0003
Bacillus phage Bam35c	Thermococcus kodakarensis	0.00116
Thermococcus sp. CL1	Thermococcus kodakarensis	0
Thermococcus sp. CL1	Methanococcus voltae	0
Thermococcus sp. CL1	Sulfolobus turreted icosahedral virus	0
Thermococcus sp. CL1	Sulfolobus turreted icosahedral virus 2	0
Thermococcus sp. CL1	Pyrobaculum oguniense TE7	0
Thermococcus sp. CL1	Invertebrate iridescent virus 6	0.001667
Thermococcus sp. CL1	Phaeocystis globosa virus	0.001667
Thermococcus kodakarensis	Methanococcus voltae	0
Thermococcus kodakarensis	Sulfolobus turreted icosahedral virus	0
Thermococcus kodakarensis	Sulfolobus turreted icosahedral virus 2	0
Thermococcus kodakarensis	Pyrobaculum oguniense TE7	0
Thermococcus kodakarensis	Phaeocystis globosa virus	0.001125
Methanococcus voltae	Sulfolobus turreted icosahedral virus	0
Methanococcus voltae	Sulfolobus turreted icosahedral virus 2	0
Methanococcus voltae	Pyrobaculum oguniense TE7	0
Sulfolobus turreted icosahedral virus	Sulfolobus turreted icosahedral virus 2	0
Sulfolobus turreted icosahedral virus	Pyrobaculum oguniense TE7	0
Sulfolobus turreted icosahedral virus	Pseudoalteromonas phage PM2	0.000089
Sulfolobus turreted icosahedral virus 2	Pyrobaculum oguniense TE7	0
Sulfolobus turreted icosahedral virus 2	Pseudoalteromonas phage PM2	0.000089
Pyrobaculum oguniense TE7	Pseudoalteromonas phage PM2	0.000031
Sputnik virophage	Organic Lake virophage	0.000667
Sputnik virophage	Phaeocystis globosa virus	0.001667
Organic Lake virophage	Phaeocystis globosa virus	0.000667
Marseillevirus	Cafeteria roenbergensis virus BV-PW1	0
Marseillevirus	Acanthamoeba polyphaga mimivirus	0.001
Marseillevirus	Ectocarpus siliculosus virus 1	0
Marseillevirus	Emiliania huxleyi virus 86	0
Marseillevirus	Invertebrate iridescent virus 6	0
Marseillevirus	Phaeocystis globosa virus	0

Marseillevirus	Paramecium bursaria Chlorella virus 1	0
Marseillevirus	Chrysochromulina ericina virus	0.000001
Marseillevirus	Pithovirus sibericum	0
Cafeteria roenbergensis virus BV-PW1	Acanthamoeba polyphaga mimivirus	0
Cafeteria roenbergensis virus BV-PW1	Ectocarpus siliculosus virus 1	0
Cafeteria roenbergensis virus BV-PW1	Emiliania huxleyi virus 86	0
Cafeteria roenbergensis virus BV-PW1	Invertebrate iridescent virus 6	0
Cafeteria roenbergensis virus BV-PW1	Phaeocystis globosa virus	0
Cafeteria roenbergensis virus BV-PW1	Paramecium bursaria Chlorella virus 1	0
Cafeteria roenbergensis virus BV-PW1	Chrysochromulina ericina virus	0
Cafeteria roenbergensis virus BV-PW1	Simian adenovirus 25	0.000093
Cafeteria roenbergensis virus BV-PW1	Pithovirus sibericum	0
Cafeteria roenbergensis virus BV-PW1	Pandoravirus salinus [ps_862]	0.000575
Acanthamoeba polyphaga mimivirus	Vaccinia virus	0.00005
Acanthamoeba polyphaga mimivirus	Amsacta moorei entomopoxvirus 'L'	0
Acanthamoeba polyphaga mimivirus	Ectocarpus siliculosus virus 1	0
Acanthamoeba polyphaga mimivirus	Emiliania huxleyi virus 86	0
Acanthamoeba polyphaga mimivirus	Invertebrate iridescent virus 6	0
Acanthamoeba polyphaga mimivirus	Phaeocystis globosa virus	0
Acanthamoeba polyphaga mimivirus	Paramecium bursaria Chlorella virus 1	0
Acanthamoeba polyphaga mimivirus	Chrysochromulina ericina virus	0
Acanthamoeba polyphaga mimivirus	Simian adenovirus 25	0.000093
Acanthamoeba polyphaga mimivirus	Human adenovirus 2	0.001333
Vaccinia virus	Amsacta moorei entomopoxvirus 'L'	0
Amsacta moorei entomopoxvirus 'L'	Ectocarpus siliculosus virus 1	0.000667
Amsacta moorei entomopoxvirus 'L'	Phaeocystis globosa virus	0.000667
Ectocarpus siliculosus virus 1	Emiliania huxleyi virus 86	0
Ectocarpus siliculosus virus 1	Invertebrate iridescent virus 6	0
Ectocarpus siliculosus virus 1	Phaeocystis globosa virus	0
Ectocarpus siliculosus virus 1	Paramecium bursaria Chlorella virus 1	0
Ectocarpus siliculosus virus 1	Chrysochromulina ericina virus	0
Ectocarpus siliculosus virus 1	Simian adenovirus 25	0.000256
Emiliania huxleyi virus 86	Invertebrate iridescent virus 6	0
Emiliania huxleyi virus 86	Phaeocystis globosa virus	0
Emiliania huxleyi virus 86	Paramecium bursaria Chlorella virus 1	0
Emiliania huxleyi virus 86	Chrysochromulina ericina virus	0
Emiliania huxleyi virus 86	African swine fever virus	0.0019
Emiliania huxleyi virus 86	Polinton-1_DR	0.00236
Emiliania huxleyi virus 86	Pandoravirus salinus [ps_862]	0.00046
Invertebrate iridescent virus 6	Phaeocystis globosa virus	0
Invertebrate iridescent virus 6	Paramecium bursaria Chlorella virus 1	0
Invertebrate iridescent virus 6	Chrysochromulina ericina virus	0
Invertebrate iridescent virus 6	Pithovirus sibericum	0
Phaeocystis globosa virus	Paramecium bursaria Chlorella virus 1	0
Phaeocystis globosa virus	Chrysochromulina ericina virus	0
Phaeocystis globosa virus	African swine fever virus	0.001667
Phaeocystis globosa virus	Polinton-1_DR	0.000089
Phaeocystis globosa virus	Pithovirus sibericum	0.000003
Paramecium bursaria Chlorella virus 1	Chrysochromulina ericina virus	0
Paramecium bursaria Chlorella virus 1	Simian adenovirus 25	0.001333
Paramecium bursaria Chlorella virus 1	Pithovirus sibericum	0.000001
Paramecium bursaria Chlorella virus 1	Pandoravirus salinus [ps_862]	0.002
Chrysochromulina ericina virus	African swine fever virus	0.0019
Simian adenovirus 25	Human adenovirus 2	0
Simian adenovirus 25	Human adenovirus 5	0
Human adenovirus 2	Human adenovirus 5	0

MOVIE S1: Three-dimensional representation of significant similarities and lineages. Clustering virus coat proteins using HOSS and sequence information only. Shades of green indicate the PRD1-Adenovirus-lineage, shades of pink the HK97-lineage, and grey the comoviruses. Our method cleanly separates the two major proposed structure-based viral lineages (PRD1-Adenovirus- and HK97-lineages), providing truly independent, purely sequence-based support for their existence. The comoviruses cluster together but clearly as their own group with no connections to other lineages, especially to the PRD1-lineage. The control protein sequences (Myosin, Globulin and NANS) associate neither with each other nor with any of the clusters. The depiction here necessarily differs from that in Figure 2 because both are low-dimensional representations of much higher-dimensional data. In both cases, there are mathematically unavoidable deformations. In order that this movie be as informative as possible, a monotone increasing function of the raw dissimilarities was used which separates the NCLDV sequences more than the function used in creating Figure 2. In both Figure 2 and this movie, lines have been drawn between the same pairs of sequences: those which were judged to be significant on the basis of two or more alignments having raw dissimilarity scores below our threshold value of 0.0025. Due to the large number of sequences involved, not all sequences are identified by labels at all times. The intention is to allow the viewer to see both the full data set at some times and also the same clustering with only representative labels at other times.
